# Chondroitin Sulfate as a Lysosomal Enhancer Attenuates Lipid-Driven Inflammation via Lipophagy and Mitophagy

**DOI:** 10.3390/md23060228

**Published:** 2025-05-27

**Authors:** Ting Sun, Huimin Lv, Huarong Shao, Xiuhua Zhang, Anqi Wang, Wei Zhang, Fei Liu, Peixue Ling

**Affiliations:** 1 School of Pharmaceutical Sciences, Cheeloo College of Medicine, Shandong University, Jinan 250012, China; sting1111@126.com; 2 Engineering Research Center for Sugar and Sugar Complex, National-Local Joint Engineering Laboratory of Polysaccharide Drugs, Key Laboratory of Carbohydrate and Glycoconjugate Drug, Shandong Academy of Pharmaceutical Sciences, Jinan 250101, China; lhmamier@163.com (H.L.); shaohuarong@sdaps.cn (H.S.); wfzxh0318@163.com (X.Z.); waq18845161375@126.com (A.W.); 3 Zhengzhou Advanced Research Institute for New Materials, Peking University, Zhengzhou 450046, China; 4 National Glycoengineering Research Center, Shandong Key Laboratory of Carbohydrate Chemistry and Glycobiology, Shandong University, Qingdao 266237, China

**Keywords:** non-alcoholic steatohepatitis, chondroitin sulfate, subcellular regulation, lysosomal acidification, lipid metabolism, mitochondrial autophagy

## Abstract

Non-alcoholic steatohepatitis (NASH), a progressive liver disease characterized by lipid accumulation and chronic inflammation, lacks effective therapies targeting its multifactorial pathogenesis. This study investigates marine-derived chondroitin sulfate (CS) as a multi-organelle modulator capable of regulating lipid metabolism, oxidative stress, and inflammation in NASH. By employing subcellular imaging and organelle-specific labeling techniques, we demonstrate that CS restores lysosomal acidification in a NASH model, enabling the reduction of lipid droplets via lysosomal–lipid droplet fusion. Concurrently, CS upregulates dynamin-related protein 1 (DRP1), driving mitochondrial terminal fission to spatially isolate reactive oxygen species (ROS) segments for mitophagy, thereby reducing ROS levels. Notably, pharmacological inhibition of lysosomal activity using chloroquine or bafilomycin A1 abolished the therapeutic effects of CS, confirming lysosomal acidification as an essential prerequisite. Collectively, these findings reveal the potential of CS as a therapeutic agent for NASH and provide critical insights into the subcellular mechanisms underlying its protective effects, thus offering a foundation for future research and therapeutic development.

## 1. Introduction

Non-alcoholic steatohepatitis (NASH), the progressive form of non-alcoholic fatty liver disease (NAFLD), has emerged as a global public health crisis, significantly contributing to cirrhosis, hepatocellular carcinoma, and liver-related mortality [1,2,3]. The pathogenesis of NASH is driven by a complex interplay of lipid dysregulation, oxidative stress, and chronic inflammation, which disrupts intracellular organelle homeostasis and perpetuates hepatocellular injury [4,5,6]. Current management strategies predominantly rely on lifestyle modifications such as weight control and exercise, yet their efficacy remains limited [7]. To date, only one drug (Rezdiffra™) has been approved for NASH treatment, underscoring the urgent need for novel therapeutic agents [8]. Existing alternative medications, including insulin sensitizers (e.g., metformin, liraglytide) and lipid synthesis inhibitors (e.g., statins), primarily target glucose regulation or lipid oxidation to ameliorate disease symptoms but fail to address the intricate crosstalk between metabolic dysfunction and oxidative damage, which are often accompanied by poor specificity and significant side effects that greatly restrict their clinical application [9,10,11].

Organelle dysfunction plays a critical role in the biological progression of NASH, primarily mediated by impaired critical metabolic organelles such as lipid droplets (LDs) [12], mitochondria [13], and lysosomes [14], leading to deregulated lipid metabolism and excessive inflammation. For instance, impaired LD–mitochondria contact sites disrupt fatty acid trafficking, exacerbating lipotoxicity and reactive oxygen species (ROS) overproduction [15,16], while lysosomal dysfunction amplifies inflammasome activation via impaired mTORC1 signaling [17]. Subcellular imaging and organelle-specific labeling techniques, with their unique high-resolution imaging capabilities, have been extensively utilized for visualizing organelle localization and interactions [16,18,19]. Despite these advances, few therapeutics have successfully regulated multi-organelle coordination to restore metabolic homeostasis, particularly in revealing spatially coordinated organelle dynamics in NASH management.

Chondroitin sulfate (CS), a sulfated glycosaminoglycan naturally abundant in connective tissues, has gained attention for its anti-inflammatory and metabolic regulatory properties [20,21,22]. Compared to terrestrial sources, marine-derived CS, particularly from shark cartilage, exhibits unique structural features [23,24]. Specifically, mammalian CS typically exhibits 4-O-sulfation (CS-A), while marine-derived CS, particularly from shark cartilage, not only presents unique 6-O-sulfation (CS-C) but is also characterized by higher abundances of di-sulfated disaccharides, such as 4,6-di-O-sulfation (CS-E) and 2,6-di-O-sulfation (CS-D). Emerging evidence indicates that marine CS mitigates hyperlipidemia-induced inflammation and oxidative damage [25,26]. Notably, CS-E has been shown to reduce hepatic triglyceride (TG) accumulation and ameliorate liver injury [27], suggesting that its unique structural features may represent a critical determinant of marine CS’s capacity to modulate lipid metabolism and oxidative stress responses. However, its subcellular mechanisms—particularly its role in orchestrating organelle crosstalk—remain unexplored. Uncovering the regulatory network of organelle interactions mediated by CS in NASH and elucidating the molecular mechanisms and biological effects of these interactions are crucial for advancing our understanding and treatment of the disease.

This study systematically evaluates marine-derived CS as a multi-organelle modulator to address the unmet therapeutic challenge of organelle crosstalk dysfunction in NASH. CS exerts its beneficial effects, including enhancing lysosomal acidification, restoring lipid metabolism, and alleviating oxidative damage, which collectively contribute to its protective role against sodium oleate (NaOl)-induced cellular damage. These findings not only underscore the therapeutic promise of CS but also provide valuable insights into the subcellular regulatory pathways involved in NASH pathogenesis, thereby paving the way for future research and potential therapeutic interventions.

## 2. Results

### 2.1. Establishment of a NaOl-Induced NASH Cell Model and Ameliorative Efficacy

#### 2.1.1. NaOl-Induced Lipid Accumulation and Oxidative Stress in HepG2 Cells

**Figure 1:**
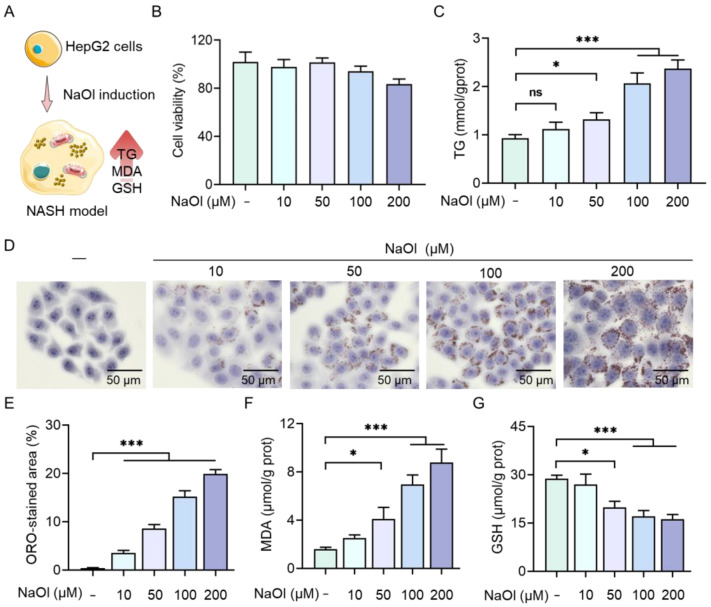
Establishment of a NaOl-induced NASH model in HepG2 cells. (**A**) Schematic of the NASH model induction protocol. (**B**) Cell viability of HepG2 cells incubated with varying concentrations of NaOl for 24 h. (**C**) Intracellular TG levels in untreated and NaOl-treated cells. (**D**) Representative ORO staining images and (**E**) semi-quantitative analysis of lipid. (**F**) MDA and (**G**) GSH levels in untreated cells and NaOl-treated cells. Data are expressed as the mean ± SEM (*n* = 3 from three independent measurements). ns refers no significant difference, *** *p* < 0.001, * *p* < 0.05 vs. untreated control.

To establish a cellular model of non-alcoholic steatohepatitis (NASH), HepG2 cells were treated with increasing concentrations of NaOl (0–200 μM) for 24 h (Figure 1A). Cell viability assessments indicated no significant cytotoxicity at concentrations ranging from 10 to 100 μM NaOl, whereas a notable reduction in viability was observed at 200 μM (*p* < 0.05; Figure 1B). Notably, TG content exhibited a dose-dependent increase, with 100 μM NaOl inducing a 2.2-fold increase compared to untreated controls (Figure 1C). Consistently, Oil Red O (ORO) staining demonstrated pronounced LD accumulation (*p* < 0.001 for 10–200 μM), as evidenced by both representative images (Figure 1D) and semi-quantitative analysis (Figure 1E).

The NASH phenotype induced by NaOl was accompanied by oxidative stress [28]. Malondialdehyde (MDA) levels, a marker of lipid peroxidation, increased by 4.3-fold in cells treated with 100 μM NaOl (*p* < 0.001; Figure 1F); glutathione (GSH) levels, reflecting cellular antioxidant capacity, showed a decline upon induction with 100 μM NaOl (*p* < 0.001; Figure 1G). Collectively, these results demonstrate that the treatment of HepG2 cells with 100 μM NaOl for 24 h effectively recapitulates hallmark NASH phenotypes—lipid accumulation and oxidative stress—without cytotoxicity, establishing a robust cellular NASH model for subsequent studies.

#### 2.1.2. CS Restores Lipid Metabolism and Alleviates Oxidative Damage in NaOl-Induced HepG2 Cells

The CS used in this study, sourced from shark cartilage, predominantly exhibits sulfation patterns characteristic of marine CS, including CS-C, CS-D, and CS-E (Supplementary Figure S1A). To validate its marine origin and ensure experimental reproducibility, comprehensive structural characterization was performed. Fourier transform infrared (FTIR) spectroscopy revealed the following key features [29]: O-H stretching vibration (3400 cm^−1^), C-H stretching vibration (2902 cm^−1^), carbonyl (C=O, 1632 cm^−1^), and N-H (1565 cm^−1^) groups, alongside sulfate signatures (S=O stretching vibration at 1238 cm^−1^) and glycosidic bond (C-O-S at 1042 cm^−1^) vibrations (Supplementary Figure S1B). Notably, peaks at 853 cm^−1^ and 821 cm^−1^ corresponded to 4-O- and 6-O-sulfation, respectively, consistent with marine CS sulfation patterns [23].

¹H and ^13^C nuclear magnetic resonance (NMR) spectroscopy were employed to further elucidate the structural features of CS (Supplementary Figure S1C,D). The ¹H NMR spectrum exhibited a distinct signal at 4.14 ppm, corresponding to GlcA-2SO_4_, which confirms the presence of 2,6-di-O-sulfation motifs [30]. Correlation analysis of proton and carbon signals via HSQC spectroscopy enabled the assignment of corresponding carbon chemical shifts. Quantification of the 4-O-sulfated to 6-O-sulfated units in CS revealed a ratio of 1.31:1, as determined by 2D HSQC NMR spectroscopy (Supplementary Figure S1E). This ratio was calculated through relative integration of the 4-sulfated GalNAc resonance (δ_H/C_ 4.61/77.1 ppm) versus the 6-sulfated GalNAc resonance (δ_H/C_ 4.24/61.04 ppm) [31]. Molecular weight was determined as 37 kDa with a narrow distribution (Supplementary Figure S1F). Sulfate content was quantified at 17.76 ± 0.33% via the barium chloride–gelatin turbidimetric method (Supplementary Figure S1G), exceeding the values reported for mammal-derived CS [32,33].

Additionally, we employed high-performance liquid chromatogram (HPLC)-based monosaccharide composition analysis to determine the components of CS. This analysis confirmed that the CS sample predominantly contained galactosamine (GalN, 53.87%) and glucuronic acid (GlcA, 34.13%), with minor contributions from glucosamine (GlcN, 5.59%), galactose (Gal, 4.39%), and mannose (Man, 2.01%)—a profile consistent with shark-derived CS standards (Supplementary Figure S2) [34]. Collectively, these findings validate the structural characteristics and marine origin of the CS sample, underscoring the rationale for selecting shark-derived CS for further mechanistic investigations.

**Figure 2:**
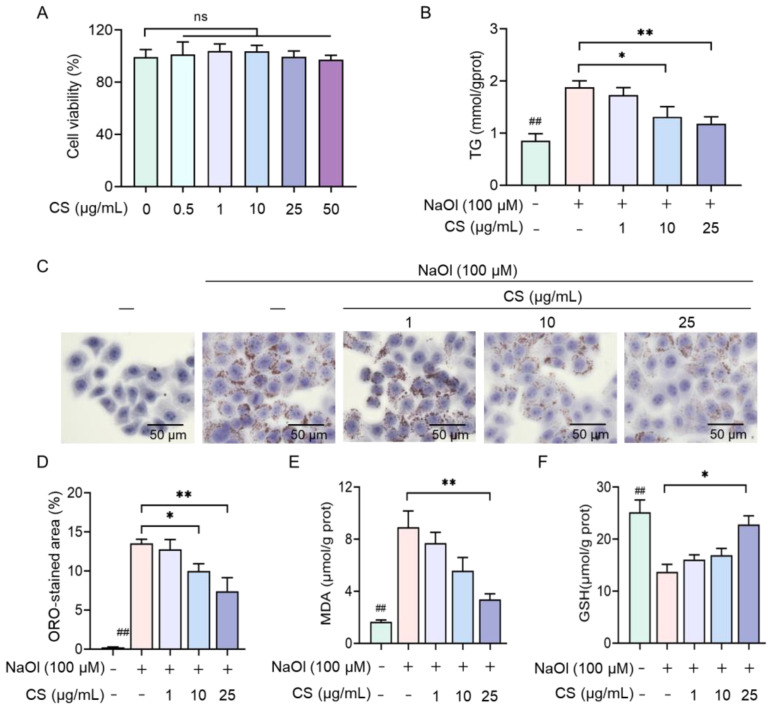
CS ameliorates NaOl-induced steatosis and oxidative stress. (**A**) Cell viability of HepG2 cells treated with varying concentrations of CS for 12 h. (**B**) Intracellular TG levels in untreated, NASH model, and CS-treated NASH model cells. (**C**) Representative ORO staining images, and (**D**) semi-quantitative analysis of lipid. (**E**) MDA and (**F**) GSH levels in untreated, NASH model, and CS-treated NASH model cells. Data are expressed as the mean ± SEM (*n* = 3 from three independent measurements). ns refers to no significant difference, ^##^ *p* < 0.01 compared to the non-treated group, ** *p* < 0.01, * *p* < 0.05 compared to the CS-treated group.

Cytotoxicity assays confirmed that treatment with 0, 0.5, 1, 10, 25, and 50 μg/mL of CS showed no significant cytotoxicity in HepG2 cells (Figure 2A), validating its suitability for subsequent functional studies. To evaluate the intervention effect of CS against NASH-related phenotypes, low (1 μg/mL), medium (10 μg/mL), and high (25 μg/mL) concentrations of CS were used in NaOL-induced HepG2 cells. Specifically, the TG contents of cells treated with 10 μg/mL and 25 μg/mL CS were significantly lower compared to the NASH model group by 30.2% (*p* < 0.05) and 37.3% (*p* < 0.01), respectively, and LD deposition was markedly decreased, as evidenced by ORO staining (*p* < 0.01; Figure 2B–D). Additionally, the high concentration of 25 μg/mL CS concurrently mitigated oxidative stress by decreasing MDA levels (*p* < 0.01), a biomarker of lipid peroxidation, and restoring GSH levels (*p* < 0.05; Figure 2E,F). These findings underscore the effectiveness of CS in alleviating NaOl-induced steatosis and oxidative damage.

### 2.2. Subcellular Regulatory Mechanisms of CS in Ameliorating the NASH Cell Model

#### 2.2.1. CS Reduces Intracellular LDs and ROS Overproduction

**Figure 3:**
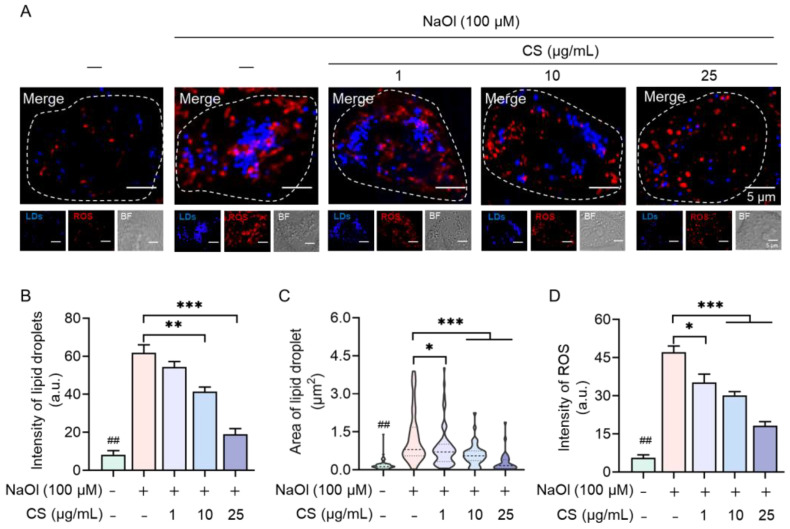
CS reduces intracellular LDs and ROS overproduction in the NASH cell model. (**A**) Confocal images of LDs (blue; λex = 405 nm) and ROS (red; λex = 540 nm). (**B**) Quantification of LD fluorescent intensity, (**C**) LD area, and (**D**) ROS fluorescent intensity. Data are expressed as the mean ± SEM (*n* = 10 from 10 cells in (**B**–**D**)). ^##^ *p* < 0.01 compared to the non-treated group. *** *p* < 0.001, ** *p* < 0.01, * *p* < 0.05 compared to the CS-treated group.

To directly visualize the effects of CS, confocal imaging was employed to visualize the accumulation of intracellular LDs and ROS in HepG2 cells induced by NaOl (Figure 3A). Induction with NaOl resulted in an increase in the number and aggregation of intracellular LDs. Following treatment with CS at concentrations of 10 μg/mL and 25 μg/mL, a significant reduction in the fluorescence intensity of intracellular LDs was observed by 32.9% (*p* < 0.01) and 69.3% (*p* < 0.001), respectively (Figure 3B). Additionally, NaOl induction suppressed lipid metabolism, leading to a notable augmentation in the area of individual LDs (*p* < 0.01). Notably, CS treatment mitigated this effect by decreasing the area of individual LDs (*p* < 0.001; Figure 3C), and the staining results for ROS indicated cellular inflammation levels, with treatment using 25 μg/mL of CS decreasing the fluorescence intensity of intracellular ROS by 61.3% (*p* < 0.001; Figure 3D). Thus, considering both the NASH-related phenotypes (steatosis, oxidative stress) and the intracellular imaging outcomes, CS at a concentration of 25 μg/mL was selected for further exploration of its subcellular regulatory mechanisms.

#### 2.2.2. CS Enhances Lysosomal Acidification to Promote Lipophagy

**Figure 4:**
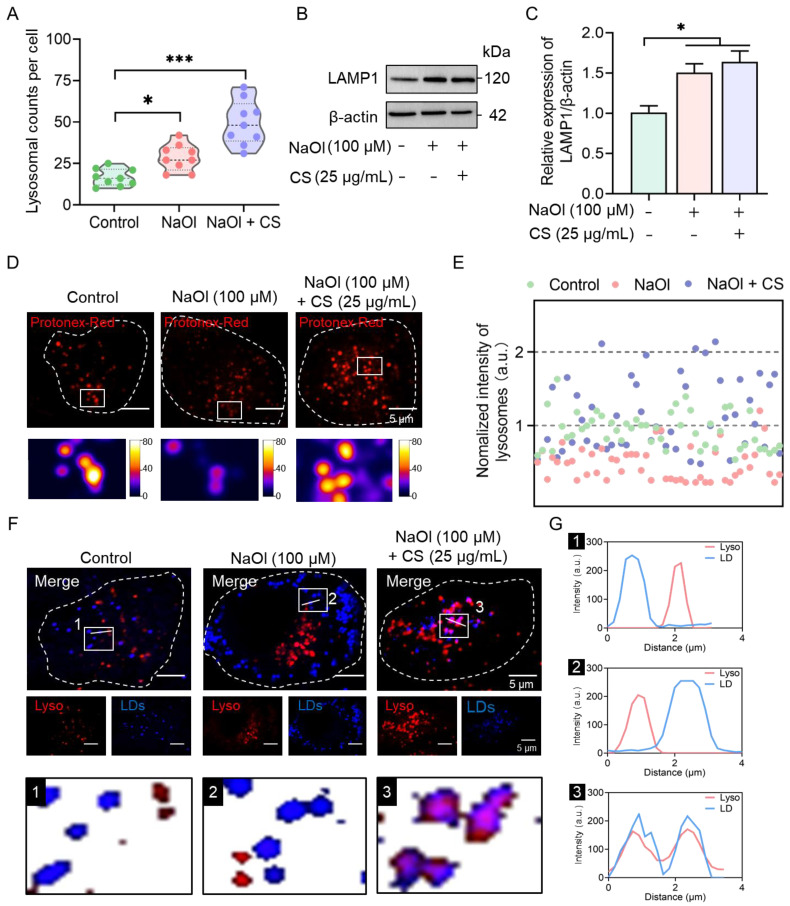
CS enhances lysosomal acidification to promote lipophagy in the NASH cell model. (**A**) Quantification of lysosomal counts in untreated and CS-treated NASH model cells. (**B**,**C**) Western blot analyses of LAMP1 protein expression. (**D**) Confocal images of lysosomal pH-sensitive probes (Protenex Red, red; λex = 580 nm). (**E**) Quantification of lysosomal fluorescent intensity from images in (**D**). (**F**) Confocal images and zoomed-in images of lysosomes (red; λex = 577 nm) and LDs (blue; λex = 405 nm). (**G**) Fluorescence intensity profiles of linear regions marked in (**F**), showing the distributions of lysosomal (red) and LD (blue) fluorescence. Data are expressed as the mean ± SEM (*n* = 9 from nine cells in (**A**), *n* = 3 from three independent measurements in (**B**)). *** *p* < 0.001, * *p* < 0.05 compared to the non-treated group or CS-treated group.

Lysosome-mediated lipophagy constitutes a crucial pathway for LD metabolism [14]. In NaOl-induced HepG2 cells, both the NaOl model group (*p* < 0.05) and the CS-treated group (*p* < 0.001) exhibited significant increases in lysosomal counts (Figure 4A) and the expression levels of lysosomal membrane-associated protein (LAMP1) (*p* < 0.05; Figure 4B,C). To determine whether the increased lysosomal abundance resulted from CS activity rather than NaOl stimulation, we performed time-course experiments with CS incubation (1, 6, 12, and 24 h) in NaOl-induced HepG2 cells. Quantitative analysis revealed a time-dependent augmentation in lysosomal numbers (Supplementary Figure S3), providing compelling evidence for CS-mediated lysosomal mobilization.

However, lysosomal functionality relies not only on their quantity but critically on their acidic microenvironment [35]. Subsequently, a pH-sensitive probe can be utilized, where a lower lysosomal pH corresponds to higher fluorescence intensity [20]. By extracting and analyzing the lysosomal signals within cells to quantify their fluorescence intensity, NaOl induction led to an increase in lysosomal pH compared to the normal group, whereas CS treatment restored lysosomal acidification, resulting in a decrease in pH (Figure 4D,E).

Additionally, spatial analysis of intracellular LDs and lysosomes revealed that LDs and lysosomes were relatively segregated in both control and NaOl-induced cells. In contrast, CS treatment induced robust colocalization between lysosomes and LDs (Figure 4F). Analysis of the fluorescence profiles of the lined regions further confirmed that CS promoted the fusion between LDs and lysosomes (Figure 4G). These results demonstrate that CS-mediated lysosomal acidification activates lipophagy, thereby alleviating NaOl-induced lipid accumulation.

#### 2.2.3. CS Activates Autophagy and Mitochondrial Fission to Eliminate ROS

**Figure 5:**
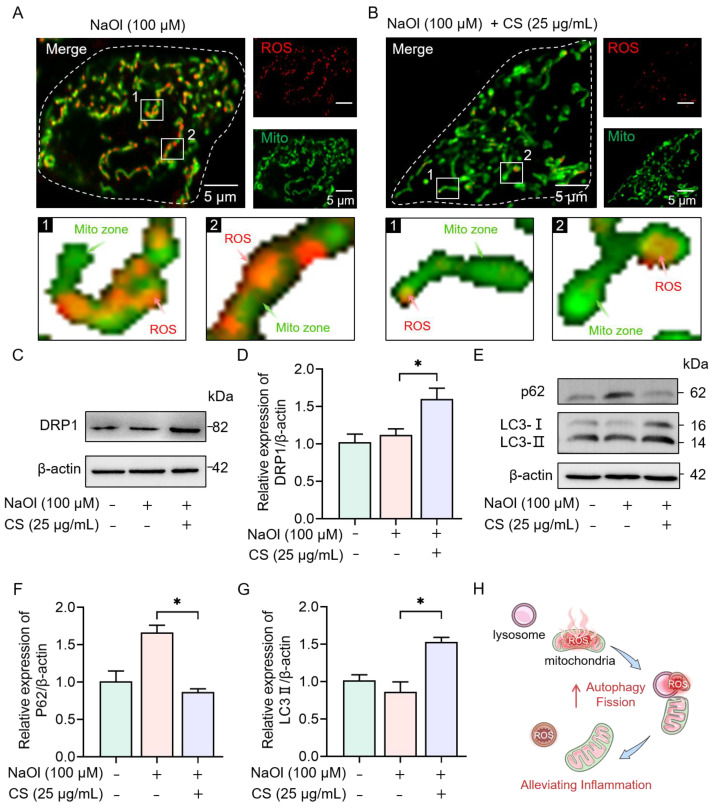
CS activates mitophagy and mitochondrial fission to eliminate ROS in the NASH cell model. (**A**,**B**) Confocal images and zoomed-in images of mitochondria (green; λex = 488 nm) and ROS (red; λex = 540 nm) in NASH model and CS-treated cells. (**C**–**E**) Western blot analyses of LC3 and p62 protein expression in untreated cells and CS-treated NASH model cells. (**F**,**G**) Western blot analyses of DRP1 protein expression in untreated cells and NASH model cells treated with CS. (**H**) Schematic representation of CS reducing inflammation in NASH model cells. Data are expressed as the mean ± SEM (*n* = 3 from three independent measurements). * *p* < 0.05 compared to the non-treated group or CS-treated group.

The results presented in Figure 3D demonstrate that CS significantly reduces ROS levels in the NASH model cells. Given that mitochondria are responsible for generating over 80% of cellular ROS [36], we performed co-staining of mitochondria and ROS to gain deeper insights into the subcellular distribution of ROS following CS treatment (Supplementary Figure S4). In the NaOl-induced group, ROS were uniformly distributed throughout the cells, as shown in Figure 5A. Conversely, CS treatment induced spatial polarization of ROS toward mitochondrial termini (Figure 5B).

Furthermore, our previous work demonstrated that CS promotes mitochondrial fission [20], leading us to hypothesize whether CS could clear ROS-enriched mitochondrial fragments by enhancing mitochondrial fission in NASH model cells. CS treatment was found to upregulate the expression of dynamin-related protein 1 (DRP1), a key protein involved in mitochondrial fission (*p* < 0.05; Figure 5C,D). This upregulation promoted terminal mitochondrial fission to compartmentalize and eliminate NaOl-induced ROS.

Critically, CS-enhanced lysosomal acidification (Figure 4E) synergistically activated autophagy, as evidenced by increased LC3- II and decreased p62 protein levels (*p* < 0.05; Figure 5E–G). This lysosomal priming ensures efficient degradation of ROS-laden mitochondrial fragments through mitophagy. These findings indicate that CS alleviates inflammation by coupling DRP1-mediated mitochondrial fission with lysosome-dependent autophagy to reduce ROS accumulation in the NASH model cells, as summarized in Figure 5H.

### 2.3. Essential Role of Lysosomal Activity in CS-Mediated Regulation of the NASH Cell Model

**Figure 6:**
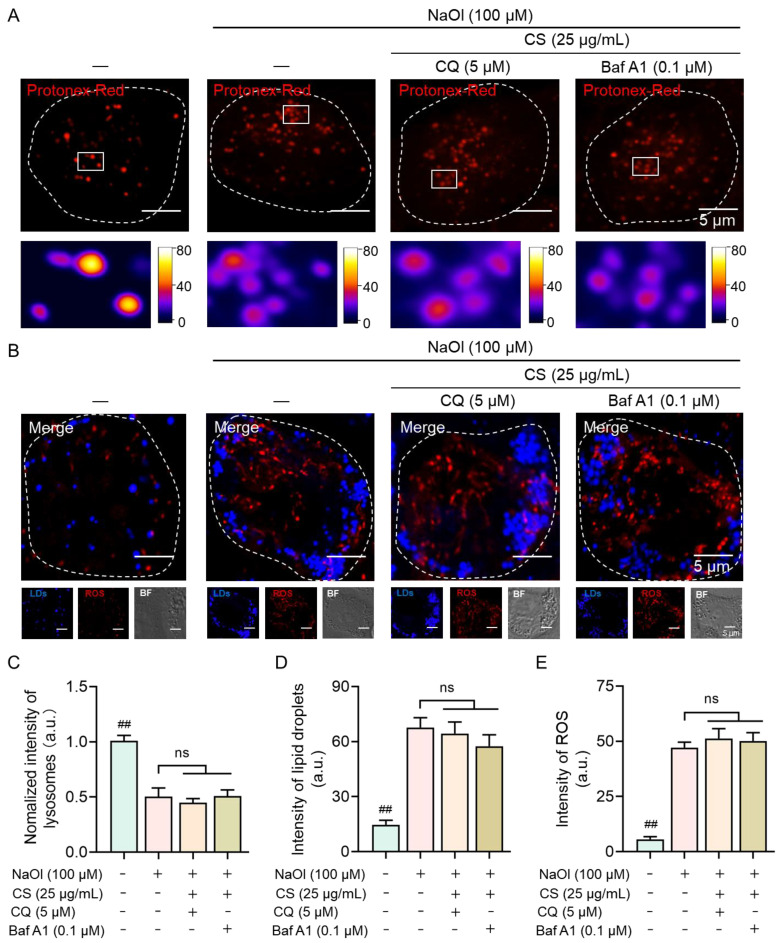
Disruption of lysosomal activity results in the failure of CS to alleviate the NASH cell model. (**A**) Confocal images and zoomed-in images of lysosomal pH-sensitive probes (Protenex Red, red; λex = 580 nm) in untreated cells and NASH model cells treated with CQ or Baf A1. (**B**) Confocal images and zoomed-in images of LDs (blue; λex = 405 nm) and ROS (red; λex = 540 nm) in untreated cells and NASH model cells treated with CQ or Baf A1. (**C**–**E**) Quantification of fluorescent intensity for (**C**) lysosomes, (**D**) LDs, and (**E**) ROS in untreated cells and NASH model cells treated with CQ or Baf A1. Data are expressed as the mean ± SEM (*n* = 10 from 10 cells). ns refers to no significant difference, ^##^ *p* < 0.01 compared to the model group.

Previous results have underscored the pivotal role of lysosomes in facilitating lipid metabolism and mitigating inflammation. To elucidate further the mechanism by which compound CS regulates NASH through lysosomal activation, we employed specific inhibitors targeting lysosomal function. Chloroquine (CQ) elevates lysosomal pH and blocks autophagosome–lysosome fusion and LC3-II degradation [37]. Additionally, Bafilomycin A1 (Baf A1), a potent inhibitor of V-ATPase, was utilized to disrupt lysosomal acidification and degradation [38]. As depicted in Figure 6A, pretreatment with CQ or Baf A1 abolished the ability of CS-induced lysosomal acidification, with lysosomal pH showing no significant difference compared to the NASH model group (*p* > 0.05; Figure 6C).

Upon confirming the impairment of lysosomal function, we next analyzed the intracellular LD accumulation and ROS levels (Figure 6B). Through quantitative assessment of fluorescence signals, it was observed that CS failed to ameliorate the elevated levels of LDs and ROS in NASH model cells pretreated with either CQ or Baf A1 (*p* > 0.05; Figure 6D,E). These results collectively demonstrate that lysosomal activity is indispensable for the regulatory effects of CS on the NASH model cells, establishing lysosomal activation as the linchpin mechanism underlying CS-mediated amelioration of NASH phenotypes.

## 3. Discussion

The pathogenesis of NASH is a multifaceted process involving lipid dysregulation, oxidative stress, and disrupted organelle dynamics [3,39,40]. The establishment of a reliable NASH cell model is pivotal for unraveling the mechanisms of this complex disease and for screening potential therapeutic interventions [41]. In this study, we successfully induced NASH-related phenotypes in HepG2 cells using NaOl, a known inducer of lipid accumulation and oxidative stress [42]. Our results indicate that treating HepG2 cells with 100 μM NaOl for 24 h effectively recapitulates the hallmarks of NASH, including lipid accumulation and oxidative stress, without causing significant cytotoxicity, ensuring the robustness of our cellular model for further studies.

Given the growing interest in natural glycosaminoglycans for the treatment of metabolic disorders, we investigated CS, derived from shark cartilage and known for its anti-inflammatory properties, for ameliorating NaOl-induced NASH-related phenotypes [23,43]. The assessment of CS’s cytotoxicity demonstrated its safety across a broad concentration range, ensuring that its beneficial effects were not confounded by cellular toxicity. Treatment with CS (25 μg/mL) significantly reduces TG content and lipid deposition (*p* < 0.01, Figure 2B–D), suggesting its potential to alleviate steatosis. Furthermore, CS treatment mitigated oxidative stress, as evidenced by decreased MDA levels and restored GSH levels (*p* < 0.01, Figure 2E,F), highlighting its antioxidant properties. These findings underscore the therapeutic potential of CS in addressing both lipid accumulation and oxidative stress, two central features of NASH.

Notably, the efficacy of CS in lipid clearance correlated with LD structural remodeling, reducing both droplet number and individual LD area (*p* < 0.001, Figure 3B,C). This suggests CS not only inhibits lipogenesis but also enhances lipid mobilization, distinguishing it from conventional lipid-lowering agents that primarily target synthesis pathways [44]. The concurrent 61.3% reduction in total ROS further emphasizes CS’s unique ability to address both metabolic and oxidative insults (Figure 3D), positioning it as a promising therapeutic candidate for NASH. While this study utilized HepG2 cells to preliminarily establish the therapeutic potential of CS in NaOl-induced NASH modeling, we recognize that immortalized cancer cells may not fully recapitulate physiological conditions. Future work will validate these findings in primary hepatocytes and in vivo models to confirm the translational applicability of CS in mitigating NASH-associated oxidative stress.

To explore the subcellular regulation of CS, we focused on lysosomal acidification and lipophagy, as well as autophagy and mitochondrial fission [45]. Lysosomes, the terminal degradative organelles, require an acidic pH (4.5–5.0) to activate hydrolases for lipophagy, a selective autophagic clearance of LDs [34]. Lysosome-mediated lipophagy is a vital pathway for LD metabolism [46,47]. In NASH, lysosomal alkalinization disrupts this process, leading to LD accumulation and triggering mitochondrial stress [48,49,50].

While high molecular weight biomolecules cannot passively diffuse into cells, prior studies have shown that glycosaminoglycans, including CS, could be endocytosed via clathrin- or caveolin-dependent pathways [51,52]. Once endocytosed, glycosaminoglycans are transported to lysosomes through the endosomal–lysosomal pathway [53]. Mechanistically, CS may act as a lysosomal stimulant by either inducing the expression of lysosomal enzymes (e.g., CTSB, CTSD) or initiating signaling pathways (e.g., mTORC1 or TFEB activation) that amplify lysosomal function, leading to increased lysosomal biogenesis and function. Our results showed that CS treatment not only increased the number of intracellular lysosomes (*p* < 0.001, Figure 4A) but also promoted their acidification (Figure 4D,E), a prerequisite for enzymatic activation and LD degradation [54]. This process aligns with evidence showing that polysaccharides can stimulate lysosomal degradation and functional activation [55]. Notably, the robust colocalization between lysosomes and LDs observed upon CS treatment suggests that CS facilitates the fusion between these organelles, thereby promoting lipophagy and clearing LDs (Figure 4F,G). These findings indicate that CS-induced lysosomal acidification drives lipophagy, thereby mitigating NaOl-induced lipid accumulation.

Concurrently, mitochondrial dysfunction in NASH fuels ROS overproduction, activating NLRP3 inflammasome and perpetuating hepatic inflammation [56,57]. Strikingly, CS induced ROS polarization to mitochondrial termini, a paradigm shift in oxidative stress management (Figure 5B). By upregulating DRP1 expression (Figure 5C,D), CS promotes terminal mitochondrial fission, isolating ROS-rich fragments for autophagic degradation [58]. This spatial compartmentalization explains the 61.3% ROS reduction (Figure 3D), suggesting a “tag-and-bag” mechanism: damaged mitochondria are first encapsulated by fission, then cleared by autophagosomes. Autophagy is critical for the degradation and recycling of damaged organelles and proteins [59], and its activation may contribute to the elimination of excess lipids and ROS in NASH cells [60]. The upregulation of autophagy markers, such as LC3-II, and the downregulation of p62 protein levels provide evidence of its role (Figure 5E–G). This polarization of ROS distribution and the subsequent elimination of excess ROS through mitochondrial fission and mitophagy contribute to reducing ROS accumulation and alleviating inflammation in NASH model cells.

To further elucidate the role of lysosomal activity in CS-mediated regulation of NASH, specific inhibitors targeting lysosomal function were employed. Pretreatment with either CQ or Baf A1 abolished the ability of CS to acidify lysosomes and impaired CS’s capacity to ameliorate the elevated levels of LDs and ROS. These results demonstrate that lysosomal activity is indispensable for the regulatory effects of CS, reinforcing the essential role of lysosomal function in CS-mediated modulation of NASH-related phenotypes.

**Figure 7:**
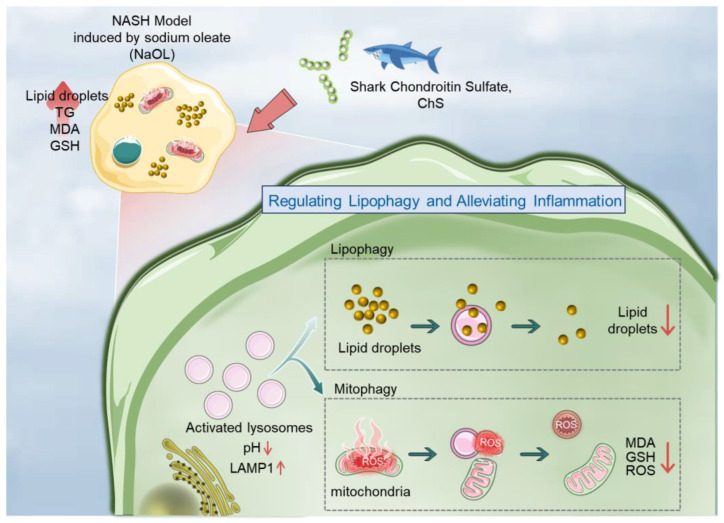
Schematic illustration of CS as a lysosomal enhancer attenuating lipid-driven inflammation via lipophagy and mitophagy.

Overall, as depicted in Figure 7, CS addresses the two central pathological features of NASH through synchronized modulation of lysosomal and mitochondrial dynamics: (1) resolving LD accumulation via restored lipophagy, and (2) mitigating ROS overproduction through spatially controlled mitochondrial fission. These findings not only delineate CS’s multi-organelle regulatory network but also contribute to the development of novel therapeutic strategies targeting organelle-associated metabolic alterations.

## 4. Materials and Methods

### 4.1. Materials

CS from shark cartilage was purchased from Aladdin Biochemical Technology (C426768, Shanghai, China). Organelle probes, including LysoTracker Red and MitoTracker Green, were purchased from Invitrogen (Eugene, OR, USA). Lipid droplet dye (Lipi-Blue) was purchased from Dojindo Laboratories (Kumamoto, Japan). Protonex-red was purchased from AAT Bioquest (Sunnyvale, CA, USA). The ROS probe (MitoSOX Red) was purchased from MedChemExpress (Shanghai, China). NaOl, ORO, Baf A1, CQ, and rotenone were purchased from MedChemExpress (Princeton, NJ, USA). The primary antibodies of rabbit anti-LAMP1, rabbit anti-DRP1, rabbit anti-LC3, and rabbit anti-actin were purchased from ABclonal (Wuhan, China). The secondary antibody of goat anti-rabbit IgG was purchased from Abcam (Cambridge, UK). Protease inhibitor cocktail, RIPA buffer, and extraction buffer were purchased from Invitrogen (Eugene, OR, USA). Fetal bovine serum (FBS), Dulbecco’s Modified Eagle Medium (DMEM), and other cell-culture reagents were provided by VivaCell (Shanghai, China).

### 4.2. Cell Culture and Treatment

HepG2 cells were purchased from Shanghai Cell Bank, Chinese Academy of Science. HepG2 cells were cultured in DMEM supplemented with 10% FBS and maintained in a humidified incubator at 37 °C with 5% CO_2_.

To assess NaOl or CS cytotoxicity, cell viability was assessed using the Cell Counting Kit-8 (CCK-8) Assay (Dojindo, Kumamoto, Japan). HepG2 cells were seeded in a 96-well plate at a density of 5 × 10^3^ cells/well in 100 μL complete DMEM and incubated for 24 h (37 °C, 5% CO₂). The culture medium was then replaced with 100 μL fresh medium containing 0, 10, 50, 100, or 200 μM of NaOl for 24 h, or containing 0, 0.5, 1, 10, 25, or 50 μg/mL of CS for 12 h. After treatment, the cells were incubated with 10 μL CCK-8 solution for 40 min at 37 °C. The absorption wavelength was measured at 450 nm using a microplate reader (Synergy H1, Thermo Scientific, Waltham, MA, USA).

To induce NASH phenotypes, HepG2 cells were treated with NaOl dissolved in DMEM. A concentration gradient (0, 10, 50, 100, 200 μM) was tested for 24 h, and 100 μM NaOl was selected for subsequent experiments.

For mechanistic investigations, low (1 μg/mL), medium (10 μg/mL), and high (25 μg/mL) concentrations of CS were selected for evaluating its intervention effects in NaOL-induced HepG2 cells for 12 h, and 25 μg/mL CS was selected for subsequent experiments.

To inhibit lysosomal activity, HepG2 cells were pretreated with 0.1 μM Baf A1 for 6 h or 5 μM CQ for 12 h prior to CS treatment.

### 4.3. Structural Characterization of CS

#### 4.3.1. FTIR Spectroscopic Analysis

The FTIR spectrum of CS was recorded on a Nicolet iS50 FTIR spectrometer (Thermo Fisher Scientific, Waltham, MA, USA). The CS sample was mixed with dried KBr powder, ground thoroughly, and pressed into a pellet for analysis. Spectra were collected in the range of 4000–400 cm^−1^ with a resolution of 4 cm^−1^ and 32 repeated scans.

#### 4.3.2. NMR Spectroscopy

CS (25 mg) was initially dissolved at room temperature using D_2_O (0.5 mL) and transferred to a 5 mm NMR tube. The 1D and 2D NMR spectra were conducted on a Bruker AVANCE 600 MHz (Rheinstetten, Germany) spectrometer to characterize the chemical structure of CS.

#### 4.3.3. Molecular Weight Measurement

The molecular weight distribution of CS was detected by gel permeation chromatography-refractive index-multiangle laser light scattering (GPC-RI-MALLS). Chromatographic separation was performed using a Shodex SB-806 column (8.0 mm × 300 mm). The CS sample was mixed with 1 mL of mobile phase (0.1 M NaNO_3_). After centrifugation at 12,000× *g* for 10 min, 100 μL of supernatant was injected into columns and subsequently eluted with the mobile phase. The flow rate was 0.4 mL/min, and the column temperature was 35 °C.

#### 4.3.4. Determination of Sulfate Group Content

The barium chloride–gelatin turbidimetric method was employed to determine the sulfate content. Standard curves were constructed using 0.04 mL, 0.08 mL, 0.12 mL, 0.16 mL, and 0.20 mL potassium sulfate solutions (6.24 mmol/L). The CS sample was hydrolyzed in HCl (5 mL, 1 mol/L) at 105 °C for 4 h. After centrifugation (8000× *g*, 10 min), the resultant supernatant was used as the test sample. Standard potassium sulfate solutions or CS samples (0.2 mL), barium chloride-gelatin solution (1 mL, 1% barium chloride, 0.5% gelatin solution), and 3% trichloroacetic acid solution (3.8 mL) were mixed and maintained (20 min, 25 °C). We measured the absorbance at 360 nm. Sulfate group contents in the CS samples were calculated using a potassium sulfate standard curve.

#### 4.3.5. Monosaccharide Composition Analysis

We prepared standard solutions by weighing 5 mg each of rhamnose, arabinose, galactose, glucose, xylose, mannose, galacturonic acid, glucuronic acid, glucosamine HCl, and galactosamine HCl, plus 10 mg fucose. We dissolved and diluted to 10 mL as stock, then diluted to specified gradients, filtered (0.22 μm), and stored. For CS samples, we hydrolyzed 5 mg polysaccharide with 1 mL 2 M TFA (121 °C, 2 h), dried under nitrogen, washed with methanol, and dissolved in sterile water. PMP derivatization was performed by mixing 0.2 mL sample/standard with 0.2 mL 0.5 M NaOH and 0.5 mL 0.5 M PMP-methanol, incubating at 70 °C for 1 h, neutralizing with HCl, extracting with chloroform, and diluting. Analysis was via HPLC (Thermo U3000, Agilent C18 column, 4.6 × 250 mm, 5 μm) using acetonitrile–phosphate buffer (pH 6.8) at 0.8 mL/min, 30 °C, 250 nm detection, and 10 μL injection.

### 4.4. Determination of TG Content

To determine the TG contents of HepG2 cells, cells were seeded in six-well plates (2 × 10⁵ cells/well) and incubated overnight. After experimental treatment, cells were trypsinized, collected, washed twice with PBS, and lysed in RIPA buffer. The lysates were directly used to measure TG content spectrophotometrically at 510 nm using a commercial assay kit (Jiancheng, Nanjing, China). Protein concentration was determined in parallel using a BCA assay to normalize TG levels.

### 4.5. Determination of MDA and GSH Levels

HepG2 cells were initially plated in six-well plates. Following the specified treatment protocols according to experimental groups, cells were trypsinized, collected, and washed. Subsequently, cells were lysed using a suitable lysis buffer containing protease inhibitors, and the lysates were centrifuged to remove insoluble debris. The supernatant was used for both MDA and GSH assays. MDA levels were quantified using the thiobarbituric acid reactive substances (TBARS) method. Absorbance was read at 532 nm. Total GSH was assayed using a glutathione colorimetric detection kit (Jiancheng Bioengineering Institute), following the manufacturer’s protocol. Protein concentration was determined in parallel using a BCA assay to normalize MDA and GSH levels to the protein content of each sample.

### 4.6. ORO Staining Assay

HepG2 cells were seeded in six-well plates at a density of 2 × 10⁵ cells/well. After treatment, cells were washed twice with PBS and fixed with 4% paraformaldehyde (PFA) for 30 min at room temperature. Fixed cells were rinsed twice with PBS and treated with 60% isopropanol for 5 min to enhance LD permeability. Cells were stained with freshly prepared ORO working solution for 15 min, protected from light. Excess stain was removed by washing three times with PBS. Nuclei were counterstained with Mayer’s hematoxylin for 1 min, followed by rinsing thrice with PBS. Images of stained cells were taken using an Olympus IX83 inverted microscope (Olympus Co., Ltd., Shinjuku, Japan). Three random fields per well were analyzed to ensure representative sampling. LD deposition was semi-quantified using ImageJ software V1.8.0 (NIH, Bethesda, MD, USA). Red-stained areas were thresholded and normalized to the total cell area.

### 4.7. Fluorescence Imaging of Cells

All fluorescence images were obtained using an LSM-980 confocal laser scanning microscope (Carl Zeiss, Oberkochen, Germany). The microscope was equipped with a 63×/1.49 numerical aperture oil immersion objective lens. Cells were cultured in 35 mm confocal laser dishes at a density of 5 × 10^4^ cells/dish. After treatment with CS and commercial probe staining, cells were washed three times with PBS and immersed in phenol red-free DMEM. Subsequently, the treated cells were observed under the LSM 980 confocal laser scanning microscope. All fluorescence images were analyzed, and their backgrounds were subtracted using ImageJ software (NIH, Bethesda, MD, USA).

### 4.8. Detection of Lysosomal pH

Cells were incubated using the lysosomal pH-sensitive probe, Protonex-red (10 μM, λex = 580 nm), at 37 °C for 30 min, followed by washing with prewarmed PBS [20]. The fluorescence intensity of this probe reflects the activity within lysosomes. Subsequently, fluorescence images were acquired on an LSM-980 confocal microscope, and the lysosomal fluorescence signals were extracted using ImageJ software to quantify the number and record the fluorescence intensity [61]. The lysosomal fluorescence intensity of the untreated control group served as the baseline standard, and the fluorescence intensity of the treated groups was normalized accordingly to assess the effects of CS or other inhibitors on lysosomal pH.

### 4.9. Quantitative Detection of LDs and ROS Level

Cells were incubated with LD dye, Lipi-Blue (10 μM, λex = 405 nm), or ROS probe, MitoSOX Red probe (10 μM, λex = 540 nm), at 37 °C for 30 min and then washed with prewarmed PBS or complete medium [62]. Fluorescence images were acquired on an LSM-980 confocal microscope. To assess the level of LDs and mitochondrial ROS, the extracted fluorescence signals were further analyzed using ImageJ software (NIH, Bethesda, MD, USA).

### 4.10. Western Blot Analysis

HepG2 cells were treated as previously described and collected [63]. Protease inhibitor, RIPA buffer, and extraction buffer were added to the centrifuged cell pellets and were then sonicated for 5 min each time, three times in total. Purified lysates were normalized using Bradford reagent. Normalized samples were mixed with LDS buffer and loaded onto 12% polyacrylamide gels. SDS-PAGE was performed at room temperature. Samples were transferred to PVDF membranes at 200 V and 4 °C. Membranes were blocked in 5% BSA/TBST for 1 h at room temperature and probed with the primary and secondary antibodies according to company guidelines. Bands were visualized using a chemiluminescence detector (ChemiDoc XRS, Hercules, CA, USA).

### 4.11. Data Analysis

Statistical analysis was performed with Prism 8 (GraphPad). An ANOVA test was used to compare the mean values of each treatment. Data are presented as mean ± SEM. The statistical comparison of results was tested with a Student’s *t*-test, with levels of significance set at ns = no significant difference, *p* < 0.05, *p* < 0.01, and *p* < 0.001. Statistical significance and sample sizes in all graphs are indicated in the corresponding Figure legends.

## 5. Conclusions

This study investigated the therapeutic potential of marine-derived CS in a NaOl-induced NASH cell model, focusing on its ability to reprogram the LDs–mitochondria–lysosome interaction network. We demonstrated that CS alleviates NASH-associated metabolic stress by synchronizing lysosomal activation, lipophagy, and mitochondrial dynamics remodeling. Through integrated subcellular analyses, we revealed that CS restores lysosomal acidification to enable LD degradation, induces DRP1-mediated mitochondrial fission to compartmentalize ROS, and optimizes autophagy for organelle quality control. These findings elucidate CS’s role as a multi-organelle modulator capable of resolving lipid-ROS crosstalk, offering a novel framework for developing multi-organelle-targeted therapies against metabolic disorders.

## Data Availability

The supporting data for this study are available within the article.

## Supplementary Materials

The following supporting information can be downloaded at: https://www.mdpi.com/article/10.3390/md23060228/s1. Figure S1: Structural characterization of CS; Figure S2: Monosaccharide composition analysis of CS; Figure S3: Time-dependent effects of CS on intracellular lysosomal counts; Figure S4: Confocal images and zoomed-in images of mitochondria and ROS in HepG2 cells.

## Abbreviations

The following abbreviations are used in this manuscript:

NASH	Non-alcoholic steatohepatitis
NAFLD	Non-alcoholic fatty liver disease
CS	Chondroitin sulfate
NaOl	Sodium oleate
MDA	Malondialdehyde
GSH	Glutathione
ORO	Oil Red O
FTIR	Fourier transform infrared spectroscop
NMR	Nuclear magnetic resonance
GPC-RI-MALLS	Chromatography-refractive index-multiangle laser light scattering
DRP1	Dynamin-related protein 1
ROS	Reactive oxygen species
LDs	Lipid droplets
LAMP1	Lysosomal membrane-associated protein 1
CQ	Chloroquine
Baf A1	Bafilomycin A1
CCK-8	Cell Counting Kit-8
