# Supplementary material for: Chondroitin Sulfate as a Lysosomal Enhancer Attenuates Lipid-Driven Inflammation via Lipophagy and Mitophagy
Source: Mar Drugs. 2025 May 27;23(6):228. doi: 10.3390/md23060228 (PMC12194273; doi:10.3390/md23060228)
Supplement: Supplementary file 1 [file marinedrugs-23-00228-s001.zip › marinedrugs-3563571-supplementary.pdf]

Supplementary Material

Chondroitin Sulfate as a Lysosomal Enhancer Attenuates Lipid-Driven Inflammation via Lipophagy and Mitophagy

Supplementary Figures

Figure S1: Structural characterization of CS.

Figure S2: Monosaccharides composition analysis of CS.

Figure S3: Time-dependent effects of CS on intracellular lysosomal counts.

Figure S4: Confocal images and Zoom-in images of mitochondria and ROS in HepG2 cells.

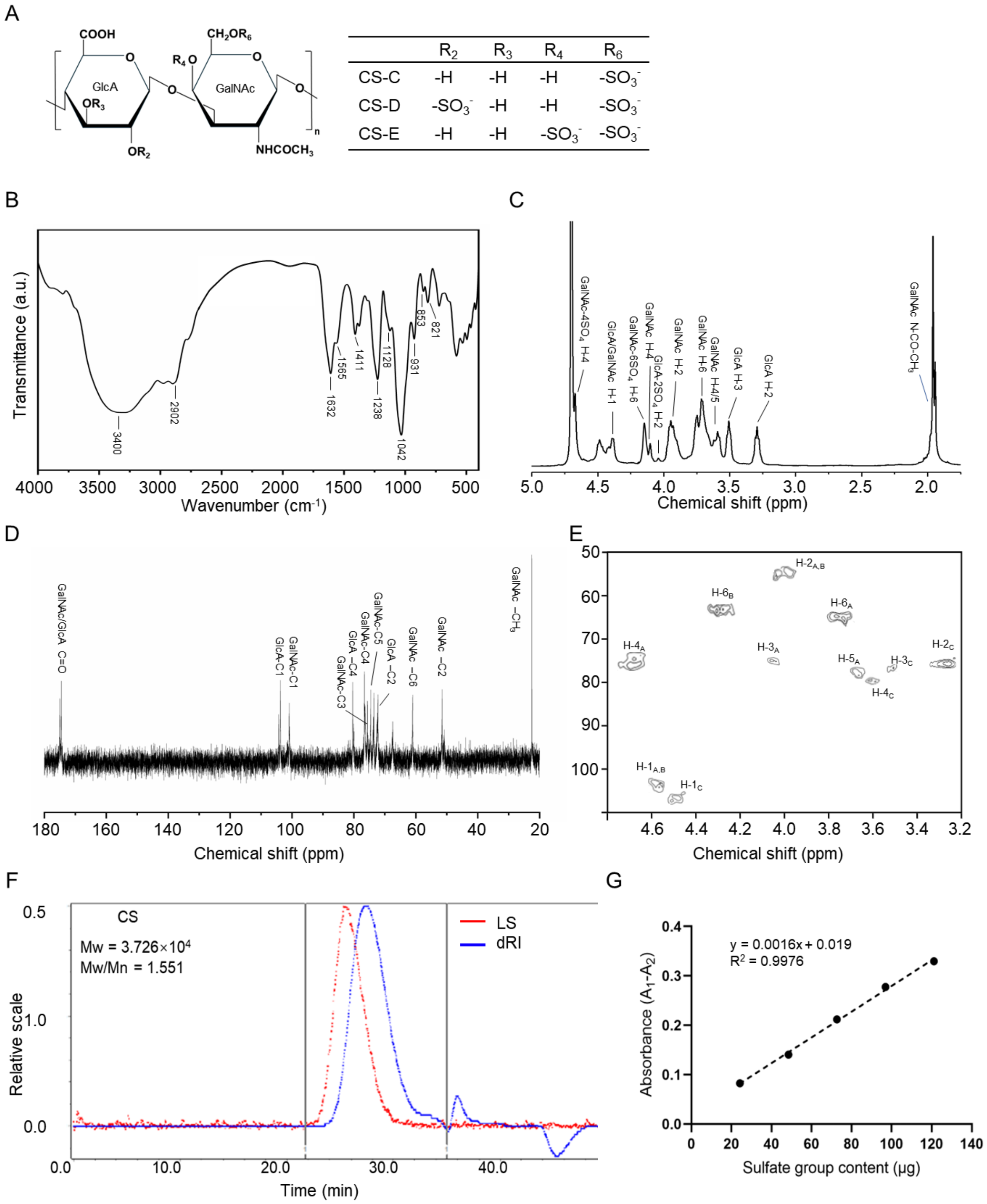

Figure S1. Structural characterization of CS. (A) Preponderance structure of CS derived from shark cartilage. (B) FTIR spectra of CS. (C) ^{1}H NMR spectra of CS. (D) ^{13}H NMR spectra of CS. (E) HSQC NMR spectra of CS (A=GalNaAc4S, B=GalNaAc6S, C=GlcA). (F) GPC-RI-MALLS spectra of CS. (G) The standard curve of potassium sulfate for calculating sulfate content.

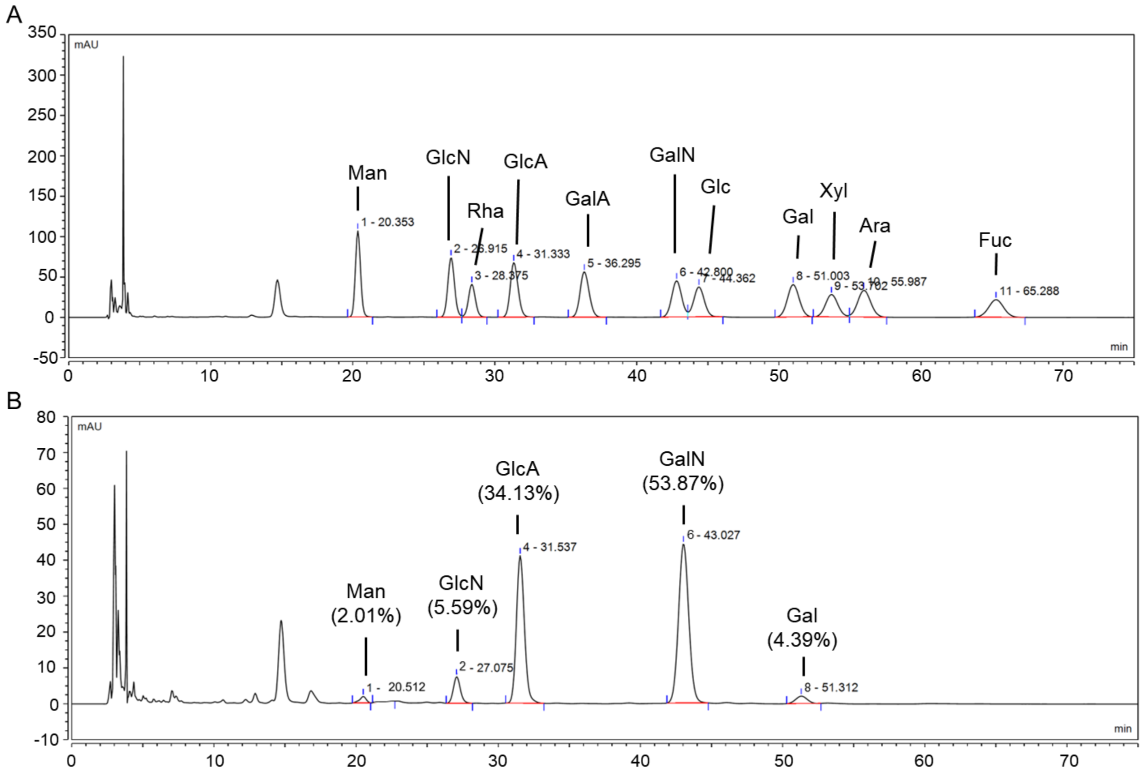

Figure S2. Monosaccharides composition analysis of CS. (A) Standard chromatograms of monosaccharides. (B) Chromatograms of CHS monosaccharid.

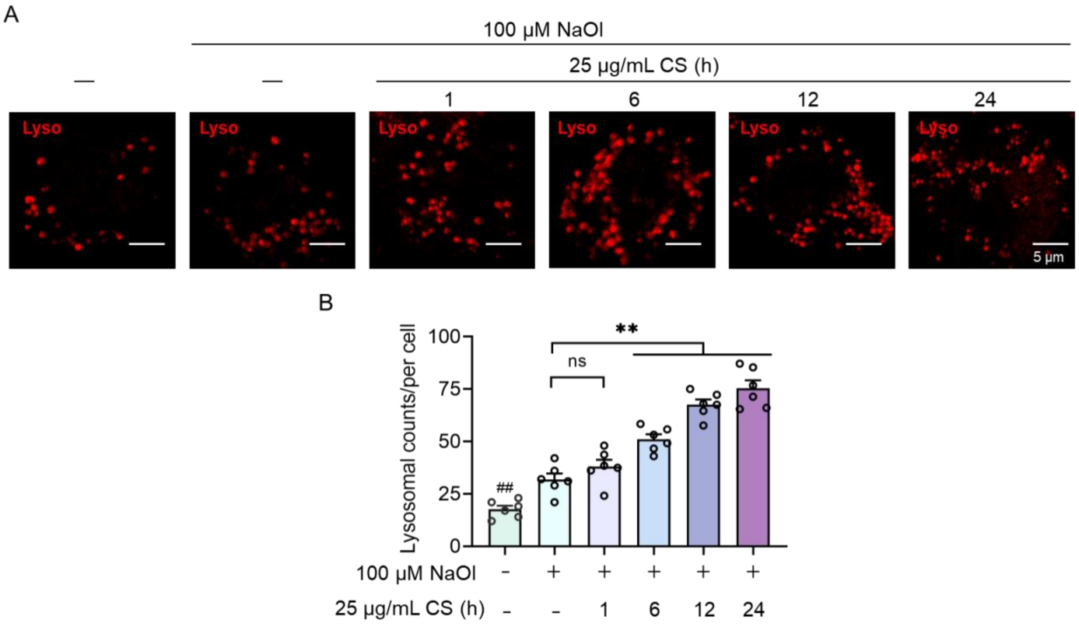

Figure S3. Time-dependent effects of CS on intracellular lysosomal counts. (A) Confocal images of HepG2 cells incubated with CS for 0, 1, 6, 12, and 24 h, stained with lysosomal probe (0.1 μM, λex = 405 nm). (B) Quantification of lysosomal counts in untreated, NASH model cells, and CS-treated NASH model cells. Data are expressed as the mean ± SEM (n = 6 from 6 cells). n.s. refers no significant difference, ## $p < 0.01$ compared to the non-treated group, ** $p < 0.01$ compared to the CS-treated group.

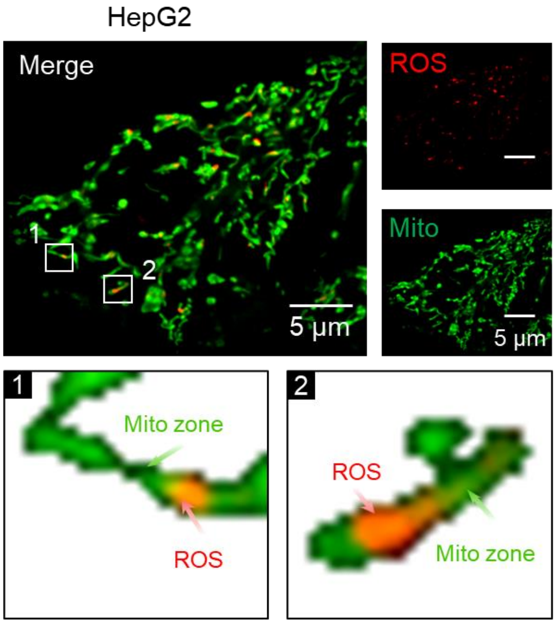

Figure S4. Confocal images and Zoom-in images of mitochondria and ROS in HepG2 cells.
